# Supplementary material for: Safety switch optimization enhances antibody-mediated elimination of CAR T cells
Source: Front Mol Med. 2022 Oct 11;2:1026474. doi: 10.3389/fmmed.2022.1026474 (PMC11285702; doi:10.3389/fmmed.2022.1026474)
Supplement: Supplementary file 1 [file Image1.pdf]

SUPPLEMENTAL INFORMATION

Safety switch optimization enhances antibody-mediated elimination of CAR T cells

Tamer B Shabaneh[1]*, Howell F Moffett[2]*, Sylvia M Stull[1], Thomas Derezes[1], Leah J Tait[2], Spencer Park[3], Stan R Riddell[1], Marc J Lajoie[2]

* T.B.S. and H.F.M. contributed equally to this study

[1]Immunotherapy Integrated Research Center, Fred Hutchinson Cancer Center, Seattle, WA

[2] Outpace Bio, Inc., Seattle, WA, USA

[3] Lyell Immunopharma, Inc., Seattle WA, USA

SUPPLEMENTAL FIGURE 1

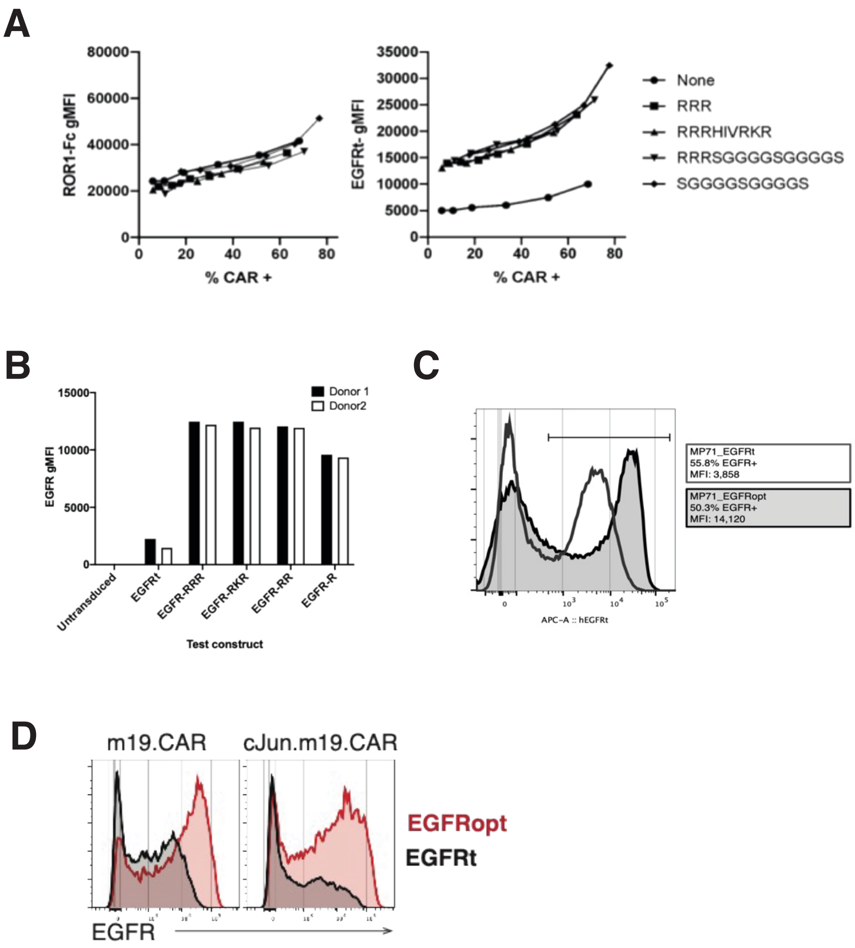

Supplementary Figure 1: Surface expression of EGFR-derived proteins

(A) Juxtamembrane domains improve surface display of EGFR across a wide range of transduction (left) without affecting surface CAR-expression (right) on CAR+ transduced T cells. (B) gMFI values of truncated EGFR molecules that include no juxtamembrane sequence (EGFRt), one arginine residue (R), two arginine residues (RR), three arginine residues (RRR), or one arginine residue swapped for lysine (RKR) encoded in a tricistronic construct. (C) Expression levels of monocistronic EGFRt (white) and EGFRopt (grey) on murine CD8^{+} T cells with similar transduction efficiencies. (D) Expression levels of EGFRt and EGFRopt on murine CD8^{+} T cells transduced with the bicistronic (left) and tricistronic (right) constructs.
